# Disseminated Histoplasmosis and Secondary Hemophagocytic Syndrome in a Non-HIV Patient

**DOI:** 10.1155/2015/295735

**Published:** 2015-08-10

**Authors:** Muhammad Kashif, Hassan Tariq, Mohsin Ijaz, Jose Gomez-Marquez

**Affiliations:** ^1^Department of Medicine, Bronx Lebanon Hospital Center, 1650 Selwyn Avenue, Suite No. 10 C, Bronx, NY 10457, USA; ^2^Division of Pulmonary and Critical Care Medicine, Department of Medicine, Bronx Lebanon Hospital Center, 1650 Selwyn Avenue, Suite No. 12 F, Bronx, NY 10457, USA

## Abstract

*Histoplasma duboisii*, a variant of *Histoplasma capsulatum* that causes “African histoplasmosis,” can be resistant to itraconazole, requiring intravenous amphotericin B treatment. Rarely, these patients do not respond to intravenous antifungal therapy, and in such cases, patients may progress to develop secondary hemophagocytic lymphohistiocytosis (HLH). We present a case of a 34-year-old male patient with sickle cell disease who presented with a 5-month history of an enlarging painless axillary mass, persistent low grade fevers, night sweats, weight loss, and anorexia. An excisional biopsy of the right axillary lymph node revealed yeast and granulomas consistent with histoplasma infection. He was started on oral itraconazole. After 4 weeks of therapy, laboratory evaluation revealed worsening anemia, thrombocytopenia, and transaminitis. Due to failure of oral therapy, he was admitted for intravenous amphotericin B treatment. During his hospital course anemia, thrombocytopenia, and transaminitis all worsened. A bone marrow biopsy was done that was consistent with HLH. His clinical status continued to deteriorate, developing multiorgan failure and disseminated intravascular coagulation. He unfortunately had a cardiorespiratory arrest after eight days of admission and passed away.

## 1. Introduction

Disseminated histoplasmosis (DH) is a chronic granulomatous disease caused by* Histoplasma capsulatum*, usually in immunocompromised patients. It typically responds to treatment with oral antifungal therapy [1, 2]. However,* Histoplasma duboisii, a variant of Histoplasma capsulatum* that causes “African histoplasmosis,” is endemic to tropical and temperate areas of sub-Saharan Western Africa and Madagascar and can be resistant to itraconazole, requiring intravenous amphotericin B treatment [3]. Rarely, these patients do not respond to intravenous antifungal therapy and in such cases, patients may progress to develop secondary hemophagocytic lymphohistiocytosis (HLH) [4, 5]. This diagnosis should be suspected in patients with persistent fever, splenomegaly, cytopenia, hypertriglyceridemia, hypofibrinogenemia, and elevated ferritin level. HLH is associated with a high mortality; hence, early recognition and initiation of treatment is crucial.

## 2. Case Presentation

A 34-year-old male presented to our outpatient clinic with painless, progressively enlarging right axillary lump that he noticed 5 months prior to presentation. His medical history was significant for sickle cell disease, and his last hospitalization had been 7 years ago for a sickle cell pain crisis. His symptoms included persistent low grade fevers, night sweats, weight loss, and anorexia. He did not have cough, dyspnea, arthritis, chest pain, or abdominal pain. The patient had no sick contacts and no contacts with animals. He had immigrated to United States from Nigeria one year prior to his presentation and had a negative tuberculin skin test. He had never smoked tobacco and had no toxic habits. He had no reported allergies. His medications included folate, iron, and multivitamin supplements.

Physical examination revealed an emaciated man. Vitals showed a fever 100.2 F, pulse rate 88/min, respiratory rate 18/min, and blood pressure 120/80 mm of hg. He was saturating 95% on ambient air. He had conjunctival pallor and palpable nontender right axillary lymphadenopathy.

Abdominal exam revealed hepatosplenomegaly. There was bilateral air entry on auscultation of lungs with no adventitious sounds. Precordial examination revealed normal heart sounds with no murmur, rub, or gallop.

**Figure 1:**
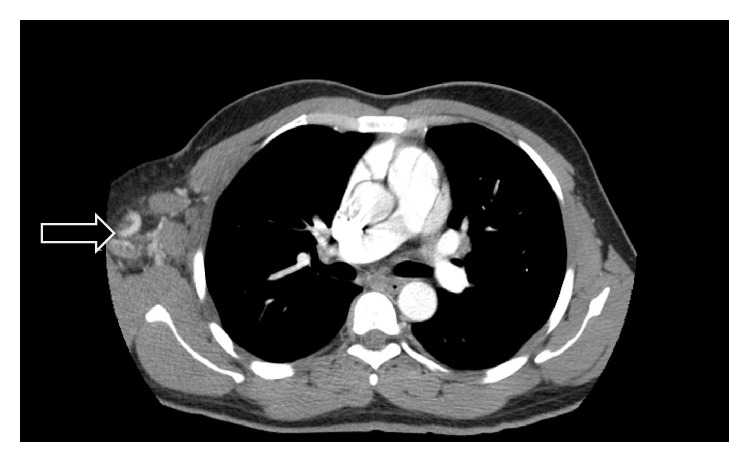
Computed tomography showing extensive right axillary lymphadenopathy (arrow).

**Figure 2:**
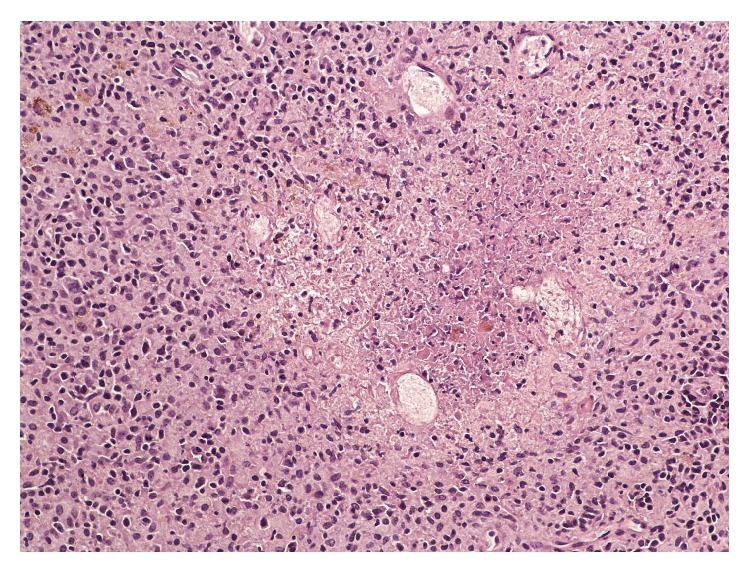
Lymph node with necrotizing granuloma comprised of necrotic center surrounded by epithelioid cells and lymphocytes.

**Figure 3:**
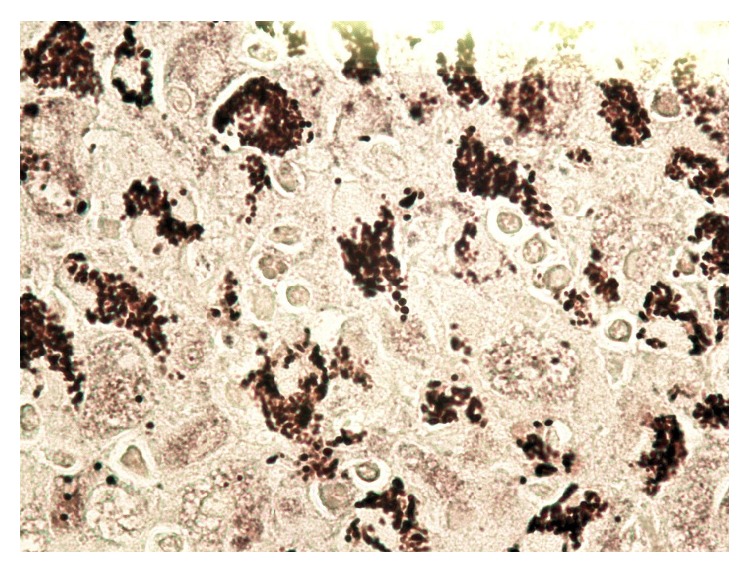
Silver stain on lymph node showing large histiocytes containing abundant yeasts (*Histoplasma*).

Computerized tomography (CT) of chest with contrast revealed multiple lung nodules, a large axillary lymph node (Figure 1), and small multiple liver masses. An excisional biopsy of the right axillary lymph node revealed yeast and granulomas consistent with histoplasma infection (Figures 2 and 3). He was started on oral itraconazole. Laboratory evaluation after 4 weeks of treatment revealed worsening anemia, thrombocytopenia, and transaminitis. Due to the failure of oral itraconazole treatment as outpatient, he was admitted for intravenous amphotericin B treatment.

His labs on initial presentation and admission and during hospitalization are summarized in Table 1. Other pertinent laboratory results are shown in Table 2.

**Figure 4:**
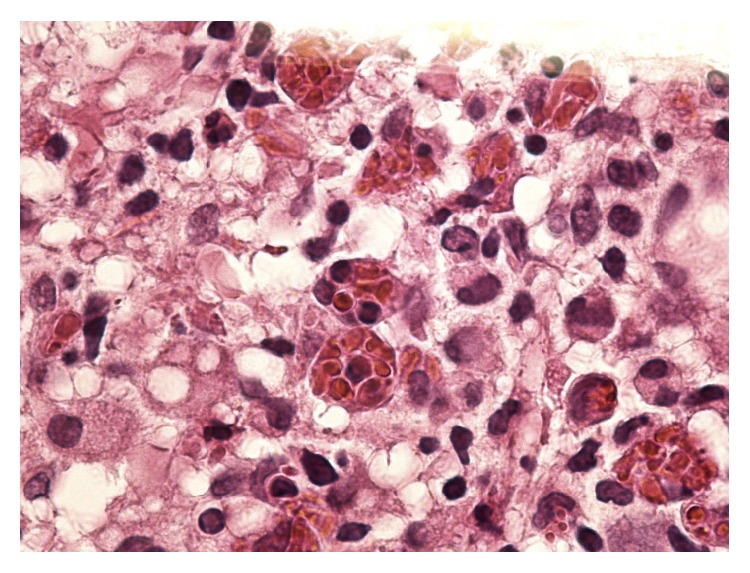
Hemophagocytic lymphohistiocytosis: bone marrow showing lymphohistiocytic infiltrate with extensive hemophagocytosis.

The patient was started on liposomal amphotericin B. Hematology was consulted for worsening anemia and thrombocytopenia, and a bone marrow biopsy was performed. His clinical status started to deteriorate on day five of admission, requiring ICU transfer for sepsis with high grade fever, tachycardia, drowsiness, and unilateral alveolar infiltrates on chest radiography. On day six, he developed septic shock and was started on vasopressors. The bone marrow biopsy revealed a lymphohistiocytic infiltrate with extensive hemophagocytosis consistent with hemophagocytic syndrome (Figure 4). The patient was started on a standard treatment protocol for HLH consisting of dexamethasone and etoposide. Unfortunately, his clinical status continued to worsen, developing multiorgan failure and disseminated intravascular coagulation. He had a cardiopulmonary arrest on day eight and passed away.

## 3. Discussion

DH is a chronic granulomatous disease caused by the dimorphic fungus* Histoplasma capsulatum* (HC). The fungus is ubiquitous and lives particularly in soil that contains large amounts of bird or bat droppings. It is endemic in the Central and Eastern United States, Central and South America, Africa, Asia, and Australia. It enters the human body through spore inhalation. Immunocompetent patients usually develop a self-limiting disease, manifested by fever, cough, and fatigue [1]. In patients with defective immune systems, the infection can become severe and spread hematogenously to other organs. Disseminated disease manifests as high grade fever, fatigue, weight loss, hepatosplenomegaly, and lymphadenopathy, mimicking the features of tuberculosis [2]. Skin lesions, pancytopenia, and anemia are more common among the immunocompromised. The disease is milder in the immunocompetent hosts with a higher cure rate [6].

Antigen detection appears to be the most sensitive rapid assay, which detects HC in 86–90% of AIDS patients [7]. The sensitivity of the urine histoplasma antigen assay in the immunocompetent patient is not well established, although it is reported to be as high as 80% compared with 82% in the immunosuppressed non-AIDS patient [8]. Polymerase chain reaction assays, blood cultures, and direct microscopic examination of specimens such as bronchial aspirates, bone marrow biopsy, or peripheral blood smear are also utilized [9], with a positive culture being the gold standard for diagnosing DH. In this case the urine and serum histoplasma antigen were negative, requiring histopathology for diagnosis.

Antifungal agents effective for treatment of histoplasmosis include amphotericin B formulations and itraconazole. Amphotericin B formulations are used for patients who have severe pulmonary or disseminated forms of histoplasmosis. Amphotericin B is generally used initially until the patient has shown a favorable response and can take an oral antifungal agent; then itraconazole is given for the remainder of the treatment course. Itraconazole given orally is preferred for patients who have mild-to-moderate histoplasmosis and as a step-down therapy after the initial response to amphotericin B. Depending on the severity of the infection and the immune status, the course of treatment can range from 3 months to 1 year. Response rate for primary therapy with itraconazole in early studies was 100% for disseminated histoplasmosis [10].

*Histoplasma capsulatum* var.* duboisii*, which causes African histoplasmosis, is endemic to tropical and temperate areas of sub-Saharan Western Africa and Madagascar and is often resistant to oral therapy with itraconazole [3]. Our patient had migrated from Nigeria, and considering the treatment failure with itraconazole, our suspicion is that he may have had the* duboisii* variant of histoplasmosis. From the onset, treatment with liposomal amphotericin B is recommended in these patients, with favorable clinical outcomes [3].

Hemophagocytic syndrome refers to a wide array of related diseases and can be primary or secondary [11]. Primary HLH syndrome involves a genetic defect caused by mutation of the perforin gene. It includes two categories: familial hemophagocytic lymphohistiocytosis and immune deficiency syndrome, which conversely also includes Chédiak-Higashi syndrome, Griscelli syndrome, X-linked lymphoproliferative syndrome type 1, Wiskott-Aldrich syndrome, severe combined immunodeficiency, and Hermansky-Pudlak syndrome. Secondary HLH is associated with a number of conditions including viral infections (29%), other types of infections (20%), malignancies (27%), rheumatologic disorders (7%), and immune deficiency syndromes (6%) [12]. HLH can occur in all age groups without predilection for race or sex [13].

HLH is believed to be the result of unrestrained macrophage activity. Macrophages serve as antigen presenting cells to lymphocytes for either direct destruction of antigens or antibody development. In the various forms of HLH, macrophages become activated and secrete cytokines. Cytokines, in turn, can cause organ damage when excreted in excessive amounts, resulting in systemic inflammatory response, immune dysregulation, and tissue damage. In addition, chronic antigen stimulation in the setting of viral infections leading to cytotoxic T-cell dysfunction has also been postulated to drive HLH. Natural killer- (NK-) cells function by directly destroying damaged or infected cells, independent of the major histocompatibility complex (MHC). Cytotoxic T-lymphocytes, while being similar to NK-cells, kill autologous cells carrying the foreign antigens associated with MHC Class I. Defects in NK-cell function may vary within the various types of HLH. Genetic forms of HLHs are due to defects in transport, processing, and function of cytotoxic granules in NK-cells and cytotoxic T-lymphocytes [14]. The ineffective antigen removal results in ongoing immune stimulation and inappropriate hemophagocytosis. The diagnosis of HLH requires fulfillment of one or both of the following criteria [11, 15]:

1. a molecular diagnosis consistent with HLH;
2. five of these eight findings:
  - fever ≥38.5°C,
  - splenomegaly,
  - peripheral blood cytopenia, with at least two of the following: hemoglobin <9 g/dL (for infants <4 weeks, hemoglobin <10 g/dL), platelets <100,000/microL, and absolute neutrophil count <1000/microL;
  - fasting triglycerides >265 mg/dL and/or fibrinogen <150 mg/dL;
  - hemophagocytosis in bone marrow, spleen, lymph node, or liver;
  - low or absent NK-cell activity;
  - ferritin >500 ng/mL;
  - elevated soluble CD25 (soluble IL-2 receptor alpha) two standard deviations above age-adjusted laboratory-specific norms.

Serum ferritin level is readily available test; with level above 10,000 *μ*g/L has a sensitivity of 90% and specificity of 96% for the diagnosis of HLH. Flow cytometry can predict the presence of a genetic defect in most cases. When available, comparison of assays measuring IFN-*γ*, IL-10, and IL-6 may be useful for distinguishing between bacterial sepsis, viral infections, and HLH in febrile patients [16]. Increased levels of serum ferritin, LDH, and liver enzymes are also valuable parameters indicating HLH [11].

There have been 18 reported cases of disseminated histoplasmosis with the hemophagocytic syndrome [4]. Twelve of them occurred in HIV-infected patients and another case involved the development of HLH associated with a vasoocclusive crisis in a patient with hemoglobin Sb+ thalassemia [17]. Infection-associated hemophagocytic syndrome can have a poor outcome. Predictors of a poor outcome include age older than 30 years, the presence of disseminated intravascular coagulation, elevated ferritin level, and anemia with accompanying thrombocytopenia [5].

Management of HLH presents many pitfalls: the condition is rare, it is not considered at onset, recognition can be difficult, and the clinical picture may be misleading [18]. Bone marrow aspiration should be considered early in the evaluation, with patients having pancytopenia, elevated ferritin levels, hypertriglyceridemia, and fever [19]. Early recognition is crucial for reasonable attempt at curative therapy to be made, given that treating an identified infection alone is not enough [15]. Life-threatening hyperinflammation, caused by excessive levels of cytokines, can be treated by corticosteroids which are cytotoxic for lymphocytes and inhibit the expression of cytokines and differentiation of dendritic cells. Most cases of infection-related HLH should be treated aggressively with standard HLH protocols that include dexamethasone, etoposide, and cyclosporine. However, delay in identification and treatment leads to fatal outcomes, as seen in this case. Duration of treatment is 8 weeks in patients with HLH who do not have an identifiable genetic defect [20]. Supportive care with prophylactic antibiotics, blood and platelet transfusions, fluid administration, and electrolyte repletion are all important steps in the treatment of HLH.

## 4. Conclusions

DH is more common in immunocompromised populations and can be complicated by atypical presentation such as HLH. A travel history to endemic areas is helpful in management. In the presence of pancytopenia, lymph node and bone marrow biopsies should be utilized early in the course of the evaluation to make the diagnosis of DH and HLH. Treatment involves a combination of antifungal and immunosuppressive therapy. Given the diagnostic difficulties and mortality associated with HLH, there should be a high index of suspicion in cases of disseminated histoplasmosis in sickle cell patients developing pancytopenia and not responding to standard treatment. Patients with infections should be monitored regularly for response since any uncontrolled infection can lead to HLH. A delay in the correct diagnosis of this complication can lead to an adverse outcome.

## Figures and Tables

**Table 1:** Laboratory values on initial presentation and admission and during hospitalization.

Labs	Initial presentation	On admission Day 1	Days 3-4	Day 8
Hemoglobin (g/dL)	12.3	8.9	6.9	4.9
Hematocrit (%)	38.2	26.6	22	13.7
WBC count (k/uL)	5.6	4.9	7.9	10
Platelet (k/uL)	283	144	92	48
Reticulocyte %		3.7	3.6	
D dimer assay (ng/mL)			4162	26206
Fibrinogen (mg/dL)			254	69
INR	1.2	1.3	2.1	4.9
Activated partial thromboplastin time (seconds)	31	31.3	34	59
LDH	632	777	1466	
Haptoglobin		2	1	
(mL/min)	87	121	87	20
Sodium (mEq/L)	138	136	145	142
Potassium (mEq/L)	4	4.4	4.2	4.4
Chloride (mEq/L)	100	97	112	106
Bicarbonate (mEq/L)	30	29	17	6
Calcium (mg/dL)	10	8.6	8.2	7.7
Phosphorus (mg/dL)		4	4.2	8.5
Glucose (mg/dL)	80	101	110	121
BUN (mg/dL)	14	29	21	36
Creatinine (mg/dL)	0.8	0.9	0.8	3
ALT (unit/L)	70	184	3050	5058
AST (unit/L)	59	214	6171	16637
Alkaline phosphatase (unit/L)	95	139	329	763
Total protein (g/dL)	7.7	5.5	6.4	3.4
Albumin (g/dL)	4.8	3.4		1.8
Total bilirubin (mg/dL)	0.7	1.2	6.3	8.1
Direct bilirubin (mg/dL)	0.3	0.6	5.1	3.1
Lactic acid (mmoles/L)		3	9.6	18
Ammonia (umole/L)			126	
Ferritin (ng/mL)			7493	
Triglycerides (mg/dL)	133	145	135	

**Table 2:** Laboratory work-up results.

Test	Result
CD4 + T help cells/UL	496 (53%)
HIV serology	Negative
CMV IgM	Negative
[HTLV-I] and [HTLV-II]	Nonreactive
EBV VCA IgM	<0.91
Serum histoplasma antigen	Negative
Urine histoplasma antigen	Negative
AFB blood culture	Negative
AFB tissue cultures	Negative
Blood cultures and urine cultures	Negative
*Blastomyces* serology	Negative
*Coccidioides* serology	Negative
*Parvovirus* serology	Negative
*Echinococcus* serology	Negative
Tissue viral culture	Negative
BAL culture	Negative
Hepatitis B surface antigen	Negative
Hepatitis B surface antibody	Positive
Hepatitis C	Negative
Serum Ceruloplasmin (mg/dL)	59
Anti-HEV IgM	Negative
Urine *Legionella* antigen	Negative
Cryptococcal antigen	Negative
Immunoglobulin G level (mg/dL)	797

## Abbreviations

ICU: Intensive care unit
CNS: Central nervous system
DH: Disseminated histoplasmosis
HLH: Hemophagocytic lymphohistiocytosis
AIDS: Acquired immunodeficiency syndrome
NK-cells: Natural killer cells.
